# Treatment of Nervous Dyspepsia by Faradism

**Published:** 1885-07

**Authors:** 


### Treatment of Nervous Dyspepsia by Faradism.

Apply the positive pole to the vertebræ promenens or between the shoulders, and the negative to the epigastrium, thus the solar plexus and its branches may be brought under the direct influence of the current. Both poles may be allowed to remain stationary for four or five minutes, the current being gradually strengthened until the stomach may be felt contorting under its influence. The negative pole should be passed transversely across the epigastric hypocondriac, umbilical, lumbar, and, indeed, over the entire abdominal region for five minutes or more. The current should be sufficiently strong to cause active contraction of the abdominal muscles. At the end of this manœuvre attach the tongue-plate to the positive conductor and instruct the patient to sit on the negative sponge, either with the nates bare or only covered by some thin fabric which may be readily moistened, so that the contact may be easily made.

Now, having removed the attachment of the positive cord from the battery, instruct the patient to put the tongue well out, and apply the tongue-plate firmly to it, then attach the cord to the battery. This will obviate an unpleasant shock which would be communicated to the tongue, were the connection made upon that organ. The current may now be gradually strengthened and allowed to continue two or three minutes. In this way the entire intestinal tract may be invigorated, and good results will certainly follow if there be no organic disease to interfere.

Sometimes the epigastrium will be tender on pressure, and the use of the negative will be attended with pain. Here we should employ the positive over the epigastrium and the negative to the spine, reversing for the purpose of diverting the soothing influence of the current; though the tongue-plate should always be used at the positive pole. the plate should be pressed firmly on the organ, as a light touch is attended by unpleasant, stinging sensations. Avoid touching the teeth with the electrode.—*California Med. Journal.*

